# Supplementary material for: A Likelihood Approach for Real-Time Calibration of Stochastic Compartmental Epidemic Models
Source: PLoS Comput Biol. 2017 Jan 17;13(1):e1005257. doi: 10.1371/journal.pcbi.1005257 (PMC5240920; doi:10.1371/journal.pcbi.1005257)
Supplement: S1 File — (TAR.GZ) [file pcbi.1005257.s014.tar.gz › HSPH_Online-SI-Revision/IPoi09/n100-mild/n100-mild_BENCH2_summary.pdf]

| peak −8 | 3w spec | 3w cum | 5w spec | 5w cum | end | 3w spec LOG | 3w cum LOG | 5w spec LOG | 5w cum LOG | end LOG |
|---|---|---|---|---|---|---|---|---|---|---|
| mean | 3805.4 | 1824.9 | 193.2 | 544.7 | 50.1 | 1. | 1. | 0.8 | 0.9 | 0.7 |
| median | 311.7 | 304. | 148.5 | 248.7 | 41.7 | 0.7 | 0.7 | 0.5 | 0.6 | 0.3 |
| quantile .1 | 129.6 | 157.8 | 53.7 | 101.9 | 11.8 | 0.4 | 0.4 | 0.2 | 0.3 | 0. |
| quantile .9 | 2539. | 2060.6 | 457. | 1123.1 | 102. | 2.3 | 2.3 | 2.1 | 0.3 | 2.4 |
| 33 | 32 | 35 | 30 | 21 | | | | | | |

| peak −4 | 3w spec | 3w cum | 5w spec | 5w cum | end | 3w spec LOG | 3w cum LOG | 5w spec LOG | 5w cum LOG | end LOG |
|---|---|---|---|---|---|---|---|---|---|---|
| mean | 58.2 | 91.7 | 48.8 | 49. | 33.3 | 0.2 | 0.3 | 0.3 | 0.2 | 0.1 |
| median | 48.6 | 87.4 | 49.3 | 41.8 | 29.2 | 0.2 | 0.3 | 0.3 | 0.2 | 0.1 |
| quantile .1 | 35.6 | 52.8 | 31.7 | 23.9 | 13.4 | 0.1 | 0.2 | 0.1 | 0.1 | 0.1 |
| quantile .9 | 89.9 | 127.6 | 64.9 | 71.9 | 53.2 | 0.3 | 0.4 | 0.5 | 0.1 | 0.3 |
| 37 | 26 | 42 | 26 | 32 | | | | | | |

| peak | 3w spec | 3w cum | 5w spec | 5w cum | end | 3w spec LOG | 3w cum LOG | 5w spec LOG | 5w cum LOG | end LOG |
|---|---|---|---|---|---|---|---|---|---|---|
| mean | 141.3 | 99.2 | 326.7 | 110. | 128.1 | 0.3 | 0.3 | 0.4 | 0.3 | 0.3 |
| median | 112.3 | 92.5 | 147.5 | 105.4 | 109.2 | 0.3 | 0.3 | 0.3 | 0.3 | 0.3 |
| quantile .1 | 60.7 | 66. | 62.4 | 59.4 | 55.8 | 0.2 | 0.2 | 0.2 | 0.2 | 0.2 |
| quantile .9 | 164.9 | 118.6 | 330.2 | 136.7 | 197.2 | 0.4 | 0.3 | 0.6 | 0.2 | 0.5 |
| 22 | 13 | 25 | 13 | 17 | | | | | | |

| peak +4 | 3w spec | 3w cum | 5w spec | 5w cum | end | 3w spec LOG | 3w cum LOG | 5w spec LOG | 5w cum LOG | end LOG |
|---|---|---|---|---|---|---|---|---|---|---|
| mean | 4013.2 | 235.2 | 1044.7 | 252.7 | 267.5 | 0.5 | 0.4 | 0.5 | 0.4 | 0.4 |
| median | 188.3 | 168.6 | 209.8 | 170.2 | 172.9 | 0.3 | 0.3 | 0.4 | 0.3 | 0.3 |
| quantile .1 | 77.9 | 81.7 | 61.8 | 83.3 | 75.9 | 0.2 | 0.2 | 0.2 | 0.2 | 0.2 |
| quantile .9 | 429.2 | 301.8 | 740.9 | 333.4 | 359.6 | 0.6 | 0.5 | 0.8 | 0.2 | 0.6 |
| 25 | 26 | 24 | 27 | 25 | | | | | | |

| peak +8 | 3w spec | 3w cum | 5w spec | 5w cum | end | 3w spec LOG | 3w cum LOG | 5w spec LOG | 5w cum LOG | end LOG |
|---|---|---|---|---|---|---|---|---|---|---|
| mean | 2431.1 | 1510.9 | 5505. | 1620.4 | 1656.5 | 0.7 | 0.5 | 0.9 | 0.5 | 0.5 |
| median | 279.2 | 197.2 | 416.8 | 209.3 | 220.3 | 0.5 | 0.4 | 0.6 | 0.4 | 0.4 |
| quantile .1 | 70.7 | 66.1 | 66.6 | 63.3 | 63.6 | 0.3 | 0.2 | 0.2 | 0.2 | 0.2 |
| quantile .9 | 3151.9 | 760.5 | 10 768.1 | 753.1 | 909.6 | 1.3 | 0.8 | 1.7 | 0.2 | 0.8 |
| 19 | 24 | 20 | 22 | 20 | {} | {} | {} | {} | {} | {} |
